# Influence of Build Angle and Layer Thickness on the Accuracy of 3D-Printed Fixed Dental Prostheses: A Systematic Review

**DOI:** 10.7759/cureus.102776

**Published:** 2026-02-01

**Authors:** Nadia El Mesbahi, Houda Moussaoui, Mouna Hamza, El Mehdi Jouhadi

**Affiliations:** 1 Fixed Prosthodontics Department, Faculty of Dentistry, University of Hassan II, Casablanca, MAR; 2 Laboratory of Community Health Epidemiology and Biostatistics, Faculty of Dentistry, University of Hassan II, Casablanca, MAR

**Keywords:** accuracy, build angle, dental 3d printing, fixed dental prostheses, layer thickness

## Abstract

The aim of this systematic review was to assess the effect of printing orientation and layer thickness on the accuracy of three-dimensional (3D)-printed fixed dental restoration. This systematic review followed the Preferred Reporting Items for Systematic Reviews and Meta-Analyses (PRISMA) guidelines and used the PICO strategy (Population/Patient, Intervention, Comparison, and Outcome). Electronic searches were carried out in July 2025 across PubMed and Scopus, followed by study selection and data extraction. The methodological quality and risk of bias of the included studies were assessed using a modified CONSORT checklist. A total of 11 studies met the inclusion criteria, all of them presenting a moderate risk of bias. The findings indicated that both printing orientation and layer thickness significantly influence the dimensional accuracy, including trueness and precision, as well as internal and marginal adaptation of 3D-printed restorations. The build angle and layer thickness influence the dimensional and fit accuracy of 3D-printed fixed dental restorations. Due to heterogeneity of the studies, an optimal combination of parameters cannot currently be established.

## Introduction and background

Digitization in dentistry has provided a significant improvement in dental practice, including temporization, definitive restorative, and prosthetic fabrication [1].

The adoption of computer-aided design/computer-aided manufacturing (CAD/CAM) technology has resulted in significant advancements in terms of accuracy and time efficiency. Three-dimensional (3D) printing is a CAD/CAM technology and allows the creation of physical parts constructed incrementally, point by point, line by line, or layer by layer, using a CAD model, facilitating the production of complex structures with high precision [2,3].

Beyond its time and cost efficiencies, this approach minimizes waste while accommodating larger components and delivering remarkable accuracy, particularly with undercuts or complex geometric designs [4,5].

The International Standard Organization (ISO 17296-2:2015) has classified 3D printing technologies into seven primary categories. Vat or VPP polymerization, specifically stereolithography (SLA) and digital light processing (DLP), represent the main technologies employed for the production of resin dental parts [6].

SLA is one of the most widely adopted 3D printing methods in dentistry due to its exceptional accuracy, resolution, and surface quality, achieved through the sequential polymerization of photosensitive resin layers. DLP shares the same photopolymerization principle but employs a different light delivery mechanism, dynamically generating a mask image and projecting it onto the resin surface using a digital micromirror device, thereby curing complete layers at once rather than tracing them point by point [7,8].

For successful dental rehabilitation, restorations must provide optimal accuracy with reduced internal and marginal gaps to ensure durable, functional, and clinically reliable outcomes [9]. Achieving optimal 3D-printed restorations depends on several factors throughout the fabrication process. Among these, the build orientation and layer thickness play critical roles in determining the accuracy, dimensional stability, and the marginal and internal fit of the restorations [9].

Accuracy involves both trueness and precision, and they are completely different terms.

Trueness evaluates alignment between the mean of test results and the true reference value, with the average RMS error serving as the standard metric for quantifying deviations between reference and test results, whereas precision describes how close multiple test results are to each other, typically expressed as the average root mean square (RMS) error variation within each test group [9].

The objective of this systematic review is to evaluate the influence of printing orientation and layer thickness on the accuracy defined by trueness and precision, and the marginal and internal adaptation as clinically relevant manifestations of the accuracy.

The null hypothesis is “print orientation and layer thickness do not affect the accuracy of 3D printed fixed dental restorations."

## Review

Materials and methods

Search Strategy

The search strategy and data acquisition were based on keywords and Boolean equations, following the Preferred Reporting Items for Systematic Reviews and Meta-Analyses (PRISMA) guidelines. The keywords used were "3D-printing", "build angle", "layer thickness", "accuracy", and "fixed dental restoration".

The search question is “Which build angle and/or layer thickness yields the highest accuracy and the most favorable marginal and internal adaptation of 3D-printed dental restorations?” 

The research question was developed using the PICO framework, with the population being 3D-printed dental restoration, the intervention being 3D-printing resin, composite, or ceramic restoration with a VAT 3D printer, the comparison involving different build angles and/or layer thickness used, and the outcome being the accuracy, internal, and marginal fit.

Data Selection

Two reviewers (E.N and H.M) independently screened records retrieved from the PubMed (MEDLINE) and Scopus databases. The searches were performed in July 2025.

The web search results were imported into reference management software Zotero. Duplicates were removed. The reviewers independently screened titles and abstracts for relevance, followed by full-text assessment of the selected studies for eligibility.

The Boolean equation for PubMed is:

(("3D printing" OR "additive manufacturing" OR "rapid prototyping" OR stereolithography OR "digital light processing" OR DLP OR SLA) AND ("dental onlay" OR "onlay restoration" OR "indirect restoration" OR "inlay" OR crown OR "fixed dental restoration" OR "dental prosthesis") AND (accuracy OR trueness OR precision OR "dimensional accuracy" OR "marginal fit" OR "internal fit" OR adaptation) AND (factor* OR parameter* OR variable* OR "printing parameters" OR "layer thickness" OR "build angle" OR "printing speed" OR "post-curing" OR "resin viscosity" OR "scanning strategy")) AND (dentistry[MeSH Terms]) AND ("2018"[Date - Publication] : "3000"[Date - Publication]) AND (english[Language])

The Boolean equation for Scopus is:

TITLE-ABS-KEY ( ( "3D printing" OR "additive manufacturing" OR "rapid prototyping" OR stereolithography OR "digital light processing" OR DLP OR SLA ) AND ( "dental onlay" OR "onlay restoration" OR "indirect restoration" OR "inlay" OR "dental prosthesis" ) AND ( accuracy OR trueness OR precision OR "dimensional accuracy" OR "marginal fit" OR "internal fit" OR adaptation ) AND ( factor* OR parameter* OR "printing parameters" OR "layer thickness" OR "build angle" OR "printing speed") ) AND PUBYEAR > 2017 AND ( LIMIT-TO ( LANGUAGE , "English" ) ) AND ( LIMIT-TO ( SUBJAREA , "DENT" ) OR LIMIT-TO ( SUBJAREA , "MATE" ) )

Eligibility Criteria

The inclusion criteria comprised studies that used vat-photopolymerization (VAT) 3D printing technology and assessed the effect of printing orientation and/or layer thickness on at least one accuracy-related outcome, including dimensional accuracy (trueness or precision) or marginal and internal fit of 3D-printed fixed dental restorations.

The exclusion criteria comprised studies about physical and mechanical properties and printed specimens intended for other dental applications, such as models, surgical guides, dies, occlusal devices, scaffolds, etc. In cases of disagreement, a third reviewer (EM.J) was involved to reach a consensus.

Bias Risk Assessment

Risk-of-bias assessment for the included studies was conducted according to the criteria of the modified CONSORT checklist for in vitro studies. The assessment encompassed 14 criteria as illustrated in Table 1. Each item that was fulfilled in the studies was designed as “YES,” while the items that failed to meet the criteria were designed as “NO”.

**Table 1:** Modified CONSORT checklist for bias risk evaluation for laboratory studies on dental materials

Authors and year	Abstract	Introduction		Methodology: (3) intervention, (4) outcomes, (5) sample size, randomization: (6) sequence generation, (7) allocation concealment, (8) implementation, (9) blinding, and (10) statistical method								Results	Discussion	funding	Protocol	Total		
																	Item	1	2a	2b	3 Intervention	4 Outcomes	5 Sample size	6 Sequence generation	7 Allocation concealment	8 Implementation	9 Blinding	10 Statistical method	11	12	13	14			
																	Al Dulaijan et al. (2024) [10]	Yes	Yes	Yes	Yes	Yes	No	No	No	No	No	Yes	Yes	Yes	No	No	8		
Alharbi et al. (2016) [11]	Yes	Yes	Yes	Yes	Yes	No	No	No	No	No	Yes	Yes	Yes	No	No	8		
Cameron et al. (2024) [12]	Yes	Yes	Yes	Yes	Yes	No	No	No	No	No	Yes	Yes	Yes	Yes	No	9		
Farag et al. (2024) [13]	Yes	Yes	Yes	Yes	Yes	No	No	No	No	Yes	Yes	Yes	Yes	Yes	No	10		
Hasanzade et al. (2023) [14]	Yes	Yes	Yes	Yes	Yes	No	No	No	No	No	Yes	Yes	Yes	No	No	8		
Metin et al. (2024) [15]	Yes	Yes	Yes	Yes	Yes	No	No	No	No	Yes	Yes	Yes	Yes	Yes	No	10		
Mou et al. (2024) [16]	Yes	Yes	Yes	Yes	Yes	No	No	No	No	No	Yes	Yes	Yes	Yes	No	9		
																	Osman et al. (2017) [17]	Yes	Yes	Yes	Yes	Yes	No	No	No	No	No	Yes	Yes	Yes	No	No	8		
Osman et al. (2025) [18]	Yes	Yes	Yes	Yes	Yes	No	No	No	No	Yes	Yes	Yes	Yes	Yes	No	10		
																	Park et al. (2019) [19]	Yes	Yes	Yes	Yes	Yes	No	No	No	No	No	Yes	Yes	Yes	Yes	No	9		
Revilla-León et al. (2025) [20]	Yes	Yes	Yes	Yes	Yes	No	No	No	No	Yes	Yes	Yes	Yes	Yes	No	10																			

A score greater than 10 indicates a low risk of bias, scores of 8-10 indicate a moderate risk of bias, and scores of 7 or fewer indicate a high risk of bias.

Assessed Outcomes

Studies can be meaningfully compared only through outcomes that quantify geometric fidelity and clinical adaptation. The principal outcome measures are divided into two complementary categories, dimensional accuracy outcomes and adaptation outcomes. Dimensional accuracy describes the overall geometric fidelity of a 3D-printed restoration, while marginal and internal gaps represent a localized assessment of the restoration’s adaptation quality. Adaptation outcomes, represented by marginal and internal adaptation, are the clinical expression of dimensional accuracy.

These outcomes are the most consistently reported across in vitro studies and therefore constitute the basis for comparison.

Thus, the assessed outcomes included dimensional accuracy, measured by trueness and precision, as well as marginal and internal adaptation, which were considered clinical manifestations of dimensional deviations.

Due to the substantial clinical and methodological heterogeneity among the included studies (materials, SLA/DLP technologies, printing parameters, types of restorations, assessed outcomes), a meta-analysis could not be performed. A qualitative synthesis was therefore adopted to describe and compare the trends observed across studies.

Results

Study Selection

**Figure 1:**
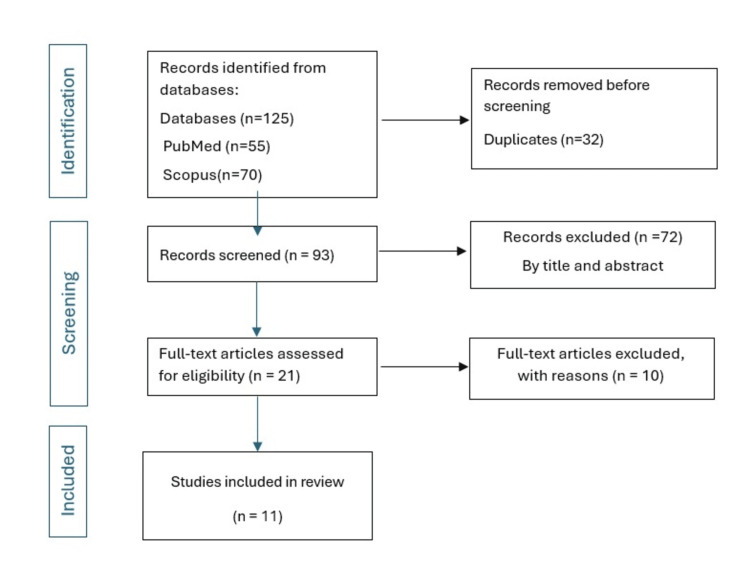
Preferred Reporting Items for Systematic Reviews and Meta-Analyses (PRISMA) flow diagram illustrating the study selection process

The web search yielded a total of 125 studies, 70 Scopus, and 55 PubMed. The duplicates were removed (n = 32). The titles and abstract screening excluded 72 studies. The number of studies assessed for eligibility was 21. A total of 11 studies were included in the systematic review. Ten studies were excluded with the following reasons: did not assess impression parameters (different build angle and layer thickness) (n = 5) ­[21-25], did not use a VAT 3D printer (n = 4) [26-29], and one study [30] did not assess dental restoration. The study selection is illustrated in Figure 1.

Bias Risk Assessment

Following the modified CONSORT statement, all 11 studies demonstrated a moderate risk of bias, as indicated in Table 1.

Characteristics of the Included Studies

The characteristics of the 11 included studies are detailed in Table 2. 

**Table 2:** Characteristics of the included studies NR: not reported, NA: not assessed

Author, year, country	Materials	Restoration type	Printing technology	Sample	Build angle	Layer thickness (µm)	Assessed outcomes	Quantitative results	Interaction effect (build angle and layer thickness)
Mou et al. (2024) [16] China	Zirconia 3Y-TZP (ADT-ZrO2-THP01-A; Shenzhen Adventure Tech Co. Ltd., China)	Single crown	DLP ADT-3D-ZP-Printer-Pro-192–50, Shenzhen Adventure Tech Co. Ltd., China.	90 samples (n = 15)	0◦, 45◦, and 90◦	30, 50	Accuracy: trueness, precision	Higher trueness: lowest RMS value observed at 90° (RMS 37,4 (4,0) / 82,8 (13, 7))	Significant interaction effect on intaglio surface trueness (p < 0.0001) -No significant effects on the marginal area or precision.
Al-Dulaijan et al. (2024) [10] Saudi Arabia	ASIGA Asiga DentaTOOTH (ASIGA, Erfurt, Germany NextDent C&B (CB) NextDent, Soesterberg, Netherlands	3-unit provisional prostheses FPD	DLP -NextDent 5,100 printer, Netherlands -Asiga MAX printer, Australia	260 specimens (130 per resin, n = 10/group)	0°, 45°, 90°	50 Post curing time (PCT): 30, 60, 90, and 120 min	Internal /marginal fit	Internal fit: ASIGA group, 0°> 45 °and 90°. Marginal fit: poorest results for the 0°/120-min PCT group (153.10 ± 61.35). NextDent group, printing orientation, and PCT did not significantly affect internal fit of restorations (P > 0.05).	NA
Revilla-León et al. (2025)[20] USA	Resin-ceramic material (Irix Max Photoshade A1-A3,5, single-use cartridges; DWS)	Molar crown	TSLA: Vat-polymerization tilting stereolithography (DFAB Chairside; DWS)	Four groups (n = 30/group)	0°, 45°, 75°, and 90°	60	Accuracy: trueness, precision	Highest accuracy at a 0° (mean ± SD RMS error: 54 ± 5 µm) and lowest at a 90° (61 ± 7 µm).	NA
Osman et al. (2025) [18] Egypt	Ceramic-filled hybrid composite material in monochromatic shade (IrixMax monochrome A2; DWS, Thiene, Italy)	Veneer for maxillary central incisor	TSLA DFAB; DWS, Thiene, Italy	Three restoration designs: window, butt-joint, incisal overlap (N = 30 per each design group). N = 10 per each build angle.	90◦, 75◦, 45◦	50	Accuracy: trueness, precision	Trueness: lower RMSE for the butt-joint veneer preparation (CL II) design (0.113 ± 0.010) mm Precision: RMSE at the 45° build angle (0.048 (±.007)) is higher relative to the 75° (0.045 (±.007)) and 90° (0 .046 (±.005)).	NA
Alharbi et al. (2016) Saudi Arabia [11]	Hybrid composite resin material (Temporis DD-1000, LOT 040725, DWS)	Full-coverage crown	SLA DW028D, DWS	-18 specimens - Two support configurations (thin and thick) for each build angle	120°, 135°, 150°, 180°, 210°, 225°, 240°, 270°	50	Dimensional accuracy	Optimal RMSE value of 0.027 mm for 90° with thin support	NA
Park et al. (2019) [19] Korea	NextDent C&B (3D systems, Soesterberg, the Netherlands) -PMMA resin blocks (Yamahachi Dental MFG, Ochigara, Japan)	Implant-supported three-unit prosthesis	DLP: D2-120 (Hephzibah, Incheon, Korea) -5-axis milling machine (IDC MILL 5X; Amann Girrbach AG, Koblach, Austria)	-10 groups (N=10/group) -Ten milled prostheses	0°, 30°, 45°, 60° and 90°	50, 100	-Marginal gap (MG) and internal gap volume (IGV)	IGV lowest at 100 µm/90° (45.5 ± 2.5 µm) and highest at 100 µm/0° (53.7 ± 2.6 µm). -MG, 100 µm/ 60°, the smallest value of 50.0±14.7 µm	Significant interaction (p = 0.001).
Hasanzade et al. (2023) [14] Iran	Acrylic‐based urethane methacrylate photopolymer resin (PowerDent Temp resin, Protech)	Full‐coverage interim crown for maxillary right first molar	DLP Digident plus	Three groups (N = 12/group)	45°	25, 50,100	- Internal and marginal fit	50 µm layer thickness: most favorable fit in all regions (mean ± SD values of 159.93 ± 11.74 µm marginal, 147.62 ± 17.57 µm axial, and 174.33 ± 21.75 µm axio-occlusal. 25 µm layer thickness: optimal fit in occlusal region (282.83 ± 53.73 µm).	NA
Osman et al. (2017) [17] Egypt	NextDent C&B (CB) (Shade A2 NextDent, Soesterberg, the Netherlands)	Full coverage crown	DLP RapideShape D30 (Rapide Shape)	NR	120°, 135°, 150°, 180°, 210°, 225°, 240°, 270°	50	Dimensional accuracy	Lowest RMSE at 135° (0.049 mm)	NA
Farag et al. (2024) Egypt [13]	Liquid photopolymer (C&B; NexDent, 3D Systems, Soesterberg, Netherlands) for DLP printer Hybrid composite resin material (Permanent crown resin; Formlabs, Somerville, MA, USA) for SLA printer	Full anatomical mandibular molar crowns	- DLP 5100; NexDent, Soesterberg, Netherlands - SLA Form 2; Formlabs, Somerville, MA, USA	- Group DLP and Group SLA (N = 30/group). -3 subgroups/ group (n = 10/subgroup)	0°, 45°, 90°	50	Marginal gap	Mean marginal gap: lower for SLA (55.6 ± 13.59 µm), smallest for 0° (48.5 ± 9.04 µm)	NA
Cameron et al. (2024) [12] Australia	LithaCon 3Y 230, a 3 mol% yttria, suspended in a photopolymer material for CeraFab - photopolymer containing Inni-Cera zirconia powder and binding colouring Material for AON ZipPro - Block of zirconia (IPS Emax ZirCAD Prime, Ivoclar)	Full coverage crown for the first mandibular right molar	Lithoz CeraFab System S65, GmbH - AON ZipPro, AON Co., Ltd. - Milling 5-axis Programat 7, Ivoclar	- 12 samples (additive manufacturing (AM)) - One sample (subtractive manufacturing (SM))	0◦ , 45◦ , 90◦	≈13 (Lithoz) ≈24 (AON)	Accuracy: trueness	At 45° for the Lithoz group: RMS of internal surface 0.03725mm and marginal surface 0.0334 mm comparable with the milled group 0.03135 mm and 0.02515 mm, respectively.	NA
Metin et al. (2024) [15] Germany	Ceramic-filled resin material (Varseo Smile Crownplus A3, Bego, Bremen, Germany) in A3 color	Full crown, table-tops, and veneers for mandibular first molar	DLP Varseo XS, Bego, Bremen, Germany	N = 10/ group	0◦, 30◦, 45◦, 60◦, 90◦	50	Accuracy: trueness and precision	90° the poorest trueness and precision across all restoration types: table-top restorations, RMSE ± SD values of 31 ± 2 µm for trueness and 13 ± 3 µm for precision. 30° optimal trueness for both crowns and table-tops: table-tops trueness and precision RMSE values of 14 ± 1 µm and 6 ± 1 µm, respectively.	NA

The included studies were highly heterogeneous in terms of materials, restoration types, VAT 3D printers, sample number/dimensions, and outcome assessment methods. Tested restorations included single crowns, three-unit provisional prostheses, veneers, and table-tops. Most studies evaluated build angles of 0°, 45°, and 90°, with additional angles reported less frequently (30° in three studies [10,15,19]; 75° in two [18,20]; and 120°, 125°, 135°, 150°, 180°, 210°, 225°, 240°, and 270° in two [11,17]). Layer thicknesses ranged from 20 to 100 µm (20, 30, 50, 60, and 100 µm). Regarding printer technology, six studies used DLP [10,14-17,19], three used SLA [11,18,20], one assessed both [13], and one compared an industrial system (CeraFab System S65, Lithoz GmbH) with a desktop system (AON ZipPro, AON Co., Ltd.) [12].

Discussion

This systematic review aimed to evaluate the influence of build angle and layer thickness on the dimensional and fit accuracy of fixed dental restorations. The null hypothesis was rejected, as different build angles and layer thickness affected the accuracy and internal and marginal fit of the 3D-printed restorations.

Most of the studies of this review focused on assessing the accuracy of 3D printed dental restorations manufactured using different printing orientations and layer thickness, alongside the evaluation of other parameters. Substantial variability in materials, printing technologies, and study methodologies limited direct comparisons.

Three studies indicated that the accuracy of dental restorations is impacted by the layer thickness [14,16,19]. Mou et al. showed that the accuracy layer thickness of 30 μm is superior to that of a layer thickness of 50 μm. For Park et al. [19], no significant difference was observed in the marginal fit of 3D-printed prostheses produced using 100 µm and 50 µm layer thicknesses. Hasanzade et al. [14] concluded that a 50 µm layer thickness provided a superior fit in all assessed regions, with the exception of the occlusal area, in which the 25 µm layer thickness yielded the most favorable results.

However, Li et al. (2019) [21] showed that internal and marginal adaptation of stereolithography-manufactured zirconia crown with a layer thickness of 25 µm is not yet ideal for clinical application. Quantitative analysis revealed a cement space of 63.40 µm in the occlusal area, 135.08 µm in the axial area, and 169.58 µm in the marginal area, which were far from the commonly accepted value of 120 µm.

Mou et al. [16] reported that the build angle has a significant influence on the accuracy of zirconia crowns produced by digital light processing technology. Thus, to ensure margin accuracy, a build angle of 0° or 45° is recommended. Regarding marginal ﬁt, Al-Dulaijan et al. [10] found that 3D-printed crowns with ASIGA showed improved outcomes at a 90° printing orientation, while those produced with NextDent performed best at a 45° orientation. Overall fit was consistently superior for NextDent resin compared with ASIGA.

Farag et al. [13] showed that 0° produces significantly the smallest marginal discrepancy, followed by 45°, then 90°.

Regarding internal fit, Al-Dulaijan et al. [10] showed that 0-degree orientation is superior to 45° and 90° orientations for the group with ASIGA resin.

Revilla-Leon et al. [20] evaluated that a 0° printing orientation yielded the greatest intaglio surface accuracy, whereas a 90° orientation produced the lowest values.

 For Alharbi et al. [11] and Osman et al. [17] (2017), the highest dimensional accuracy was observed with a build angle of 120° and 135°, respectively. Another study by Yu et al. [31] used a 3D SLA printer and assessed the intaglio surface at the trueness of the intaglio surface and the marginal integrity of the interim crown with a layer thickness of 100 µm. The study found that the intaglio surface is accurately reproduced between 150° and 210° of build angle.

Ryu et al. [32] used a DLP printer, with the layer thickness set to 50 μm, and the optimal build angles were 150° and 180°.

Metin et al. [15] demonstrated that both trueness and precision were lowest at a 90° build orientation. Although Cameron et al. [12] observed that printing trueness showed a progressive decline, with the smallest yet significant deviations observed in samples printed at 0°, followed by the 45° and 90° orientations. Alghauli et al. [9] showed that the angles 180°, 30°, 45°, and 60° presented comparable marginal gaps, while 90° and 120° build-up angles resulted in significantly higher marginal gaps than the 180° build-up angle.

Other studies of 3D printed models showed the same results. ElShebiny et al. [33] reported that the highest accuracy was observed in horizontally oriented SLA models produced with 50 µm and 100 µm layer thicknesses, whereas models printed at 25 µm showed comparatively lower accuracy and those fabricated in a vertical orientation at all thicknesses.

Short et al. [34] reported analogous outcomes, showing that vertical build orientation led to the lowest accuracy, while 0° and 20° orientations yielded superior model accuracy.

## Conclusions

This systematic review demonstrates that build angle and layer thickness affect the dimensional and fit accuracy of 3D-printed dental restorations, as reflected by variations in trueness, precision, and marginal and internal adaptation.

Variations in materials, printing technologies, build angles, and other factors, such as post-curing procedures, as well as the location and orientation of supports, make it difficult to compare the findings of this study and those in the existing literature.

Careful selection of the parameters of 3D printing is mandatory to yield optimal results.

Due to heterogeneity in study designs, materials, printing technologies, and outcome assessment methods, an optimal combination of parameters cannot currently be established.

Clinical studies are required to investigate the survival rate of 3D printed dental restoration.
